# Smoked, smokeless, and poly-tobacco use during pregnancy in relation to infant mortality in Cambodia: Findings from a nationwide sample

**DOI:** 10.18332/tid/191718

**Published:** 2024-09-23

**Authors:** Jiahao Peng, Anne Berit Petersen, David Shavlik, Daliao Xiao, Daravuth Yel, They Kheam, Pramil N. Singh

**Affiliations:** 1 School of Public Health, Loma Linda University, Loma Linda, United States; 2 School of Nursing, Loma Linda University, Loma Linda, United States; 3 Loma Linda University Cancer Center, Loma Linda, United States; 4 Lawrence D. Longo MD Center for Perinatal Biology, Department of Basic Sciences, Loma Linda University School of Medicine, Loma Linda, United States; 5 Cambodia Tobacco Free Initiative, World Health Organization, Phnom Penh, Cambodia; 6 National Institute of Statistics, Phnom Penh, Cambodia

**Keywords:** maternal tobacco use, smokeless tobacco, infant mortality, tobacco cessation, reproductive health

## Abstract

**INTRODUCTION:**

Maternal cigarette smoking during pregnancy is an established risk factor for adverse maternal, fetal, and infant outcomes. In contrast, maternal smokeless tobacco use (i.e. e-cigarettes, snus, betel quid, iqmik) during pregnancy has a more complex risk profile due to its potential use as a smoking cessation aid or to reduce the harm from smoking tobacco. The overall aim of this study was to investigate the association between smoked, smokeless, and poly-tobacco (smoked + smokeless) use during pregnancy and infant mortality, in a national sample of women in Cambodia.

**METHODS:**

The study used data from the National Adult Tobacco Survey of Cambodia (NATSC) that employed sampling methods and tobacco survey items from the CDC Global Adult Tobacco Survey but also included a supplement on reproductive health and birthing history. We selected 5342 women of the NATSC who reported complete data on at least one pregnancy, and our unit of analysis was the 15998 pregnancies from these women. We conducted a multivariable logistic regression to relate tobacco use to infant mortality. Taylor linearized variance estimators were used to account for clustering by sampling unit and mother.

**RESULTS:**

We found that smokeless tobacco in the form of a betel quid was the most common form of tobacco used during pregnancy. In multivariable logistic regression, we found increased odds of infant death for all tobacco use categories (smoked, smokeless), but that the strongest effects were seen for habits that included smokeless tobacco (relative to never use of tobacco in any form): exclusive use of smokeless tobacco (adjusted odds ratio, AOR=2.08; 95% CI: 1.15–3.76), and poly-tobacco use (AOR=5.68; 95% CI: 1.03–31.46). In more detailed analyses that considered the composition of the betel quid (tobacco, areca nut/leaf, slaked lime), we found that even chewing of tobacco leaves with no processing or additives was associated with a three-fold increase in odds of infant death relative to a never user (AOR=3.05; 95% CI: 1.45–6.45).

**CONCLUSIONS:**

We found that even among those pregnant women who limited their nicotine habit to chewing tobacco leaves with no processing or additives, there remained higher odds of fetal or infant death from that pregnancy.

## INTRODUCTION

Maternal cigarette smoking during pregnancy is an established risk factor for adverse fetal and infant outcomes of that pregnancy that include mortality, low birth weight, preterm birth, and a wide range of developmental outcomes observed at birth or evident in the epigenome^1-3^. This burden from adverse birth outcomes is compounded by the effect of maternal cigarette smoking during pregnancy on the excess risk of maternal morbidity during and after the pregnancy^2^. Globally, maternal cigarette smoking during pregnancy tends to occur among low-income women from health disparity populations (i.e. rurality, race/ethnicity) with barriers to adequate prenatal and preventive care^4,5^.

Maternal smokeless tobacco use (i.e. e-cigarettes, snus, betel quid, iqmik) during pregnancy has a more complex risk profile according to the scientific literature^6^. The perceived safety or reduced harm of smokeless tobacco use during pregnancy has led to the use of smokeless tobacco as either a smoking cessation aid or as a normalized, culturally acceptable form of tobacco use for reproductive age and/or pregnant women. In recent trials in the UK, e-cigarettes have been shown to be slightly more effective than nicotine replacement therapy (prolonged abstinence of 6.8% versus 3.6%, p=0.02) in helping pregnant smokers quit their habitual smoking of combustible cigarettes^7^. In Norway and Sweden, pregnant smokers continue, in a self-directed manner, to use chewing tobacco in the form of ‘Swedish snus’ during pregnancy while decreasing their rate of smoking cigarettes^8,9^. Among Alaskan native women, where up to 79% of women use smoked or smokeless tobacco (iqmik) during pregnancy, there is a perception that smokeless tobacco use in the form of chewing a traditional tobacco product (iqmik) is safer^10^. In the Western Pacific Region, traditional medicine beliefs in rural Asia promote not only the belief in the safety of smokeless tobacco in the form of betel quid (tobacco, areca nut, areca leaf, slaked lime) but also the belief that this chewing tobacco product can help with pregnancy-related symptoms (morning sickness)^11-14^.

To estimate the risk attributable to e-cigarettes, the Pregnancy Risk Assessment Monitoring System (PRAMS) cohort followed up with 79176 pregnant women in the US and reported in 2021 that exclusive e-cigarette use during pregnancy was as harmful as exclusive use of combustible cigarettes – either product producing about twice the risk (relative to no maternal use of tobacco in any form) of adverse neonatal outcomes such as preterm birth and low birth weight^15^. A systematic review of 18 cohort studies that tested the association between maternal snus use and adverse neonatal outcomes found a pooled estimate from over one million pregnant women indicating an increased risk of neonatal apnea, stillbirth, premature birth, and oral cleft malformation^16^. A systematic review of eight studies of prenatal betel quid use and infant outcomes found that it was associated with a significant 75% increase in odds of low birthweight^17^.

The overall aim of the present study was to investigate the association between smoked tobacco, smokeless tobacco, and poly-tobacco use and infant mortality, in a national sample of reproductive-age women in Cambodia. Our study leverages 20 years of tobacco research capacity building by the Fogarty/NIH grants that have partnered with the National Institute of Statistics (Ministry of Planning, Cambodia), National Center for Health Promotion (Ministry of Health, Cambodia), WHO Cambodia, and Loma Linda University in a consortium to conduct nationally adult tobacco surveys of Cambodia (NASTC) during 2006–2021^18^. Since 2012, these surveys have been modeled after the CDC Global Adult Tobacco Survey format. The 2012 NATSC included a special survey supplement on tobacco use during current and past pregnancies and was used for this study. Our specific aims are as follows: 1) To examine whether maternal smokeless tobacco use with betel quid ingredients is associated with the risk of infant death; 2) To examine whether maternal smokeless tobacco use without betel quid ingredients is associated with risk of infant death; and 3) To examine whether poly-tobacco use is associated with risk of infant death.

## METHODS

The methods of the National Adult Tobacco Survey of Cambodia (NATSC) 2012 have been previously described^13,19^. In this report, we describe the methods we used to study reproductive age (15–49 years) women of NATSC 2012 who responded to both the survey and a special survey supplement on reproductive health and birthing history.

### Study population

NATSC 2012 was designed to be a nationally representative tobacco survey of adults in Cambodia that was implemented by the National Institute of Statistics (NIS) in the Ministry of Planning of Cambodia. A sample of 15615 adults (aged ≥15 years) was selected from 6294 households from all provinces. Similar to all previous national health surveys, the NIS surveys excluded institutional households (i.e. such as military barracks, prisons, hospitals, and residents of temples). The NATSC sample was assembled using a stratified, multistage cluster sampling approach in which the 2008 Cambodia General Population Census was used as a sampling frame. The approach stratified the country into 17 distinct census-derived survey domains that consisted of provinces or groups of similar provinces. Within each domain, 25–26 primary sampling units (PSU) were selected as villages or comparable urban units for a total of 437 PSUs. Enumeration areas were designated as blocks of households and became the next sampling stage. Trained NIS survey personnel implemented all enumeration and surveying of the selected households.

For this report, we selected the 5731 women from NATSC who had reported at least one pregnancy during their lifetime. From this sub-sample, we excluded 389 women who had missing data on either the pregnancy outcome (i.e. death up to a year after delivery) or smoking status. This produced a final sample of 5342 women who reported outcomes from 15998 pregnancies that occurred during their lifetime.

Written informed consent was obtained from each participant, and the protocols for the 2012 NATSC national survey and its sub-studies were approved by the Institutional Review Board of Loma Linda University (#5170182).

### Questionnaire

*Tobacco and health items*

The NATSC items on tobacco were adapted from the CDC Global Adult Tobacco Survey (GATS). They included well-known modules on tobacco use, tobacco cessation, secondhand smoke exposure, economics, media exposure, and knowledge and attitudes. GATS items on smoked tobacco were adapted to the Cambodian experience to include manufactured cigarettes, hand-rolled cigarettes sold as a rural product, hand-rolled cigarettes by the smoker, pipe, waterpipe, and cigars. GATS items on smokeless tobacco that was chewed were adapted to the Cambodian experience to include smokeless tobacco chewed with no additives, smokeless tobacco with betel quid additives (areca leaf, areca nut, slaked lime), betel quid additives (areca leaf, areca nut slaked lime) chewed without smokeless tobacco use.

The GATS items on smoked and smokeless tobacco were administered with picture cards to help the subjects report the specific tobacco product they used and estimate the amount of tobacco used. This method was validated in a previously described sample of 201 subjects in rural Cambodia, where questionnaire and picture card administration were compared to salivary cotinine testing. The findings demonstrated an 87% sensitivity, 94% specificity, and 93% positive predictive value in detecting cotinine levels >10 ng/mL, indicating a high accuracy. Moreover, the validation also revealed significant positive correlations with the number of cigarettes smoked and the amount of tobacco chewed, as measured by a pictogram^20^.

*Reproductive health supplement*

Reproductive-age women (15–49 years) were administered a special supplement asking about their tobacco use behaviors during current and past pregnancies. The supplement also included items on a comprehensive lifetime birthing history for all parous women, detailing maternal age at birth, child mortality before one and five years of age, and the current vital status of each child. Some of these items were adapted from the birth history section of the Demographic and Health Survey developed for Cambodia^21^.

*Questionnaire-derived tobacco use definitions*

We defined smoked, smokeless, and poly-tobacco use as follows.

Smoked tobacco use

We adapted the GATS survey items on the Smoked Tobacco section of the 2012 GATS template (CDC) to include the forms of tobacco use that are found in Cambodia, such as cigarettes, cigars, pipes, and waterpipes. For clarity, we combined validated picture cards of typical smoked tobacco products with the item administration. Picture cards were developed from qualitative studies of tobacco use in rural Cambodia, and their use in a survey was validated by salivary cotinine^20^.

Smokeless tobacco use

We adapted the GATS survey items on Chewing Tobacco to include the forms of tobacco use that are found in Cambodia and included betel quid with chewing tobacco (rural and/or self-made product), chewing tobacco without betel quid (rural and/or self-made product), chewing tobacco from a tin (manufactured product). For clarity, we combined validated picture cards of chewing tobacco products with item administration. The picture card series also included a choice of betel quid products (areca nut, betel leaf, slaked lime) without chewing tobacco. Similar to the smoked tobacco section, picture cards for chewing tobacco were developed during qualitative studies of tobacco use in rural Cambodia, and their use on a survey was validated by comparison with salivary cotinine tests^20^.

Poly-tobacco use

Poly-tobacco use was defined as subjects indicating any use of both smoked and smokeless tobacco.

*Maternal tobacco during pregnancy measures*

The GATS Template items we used allowed us to classify smoked, smokeless, and poly-tobacco by temporal domains of current daily use, former daily use, age at initiation, and age at cessation for former daily users. These items were used to determine tobacco use during the life course birthing history of each parous mother, where we had data available for maternal age at birth and the birth outcome.

### Statistical analysis

The unit of analysis was an individual pregnancy and produced a sample of 15998 pregnancies. To account for the stratified multi-stage cluster sample, we considered the provincial domain as the strata variable and accounted for clustering by village/urban unit, enumeration area, and mother, by computing variances around means, proportions, and regression coefficients using a Taylor linearized variance approach that used a variance inflation factor to account for within-cluster homogeneity^22^.

Descriptive analyses included computing means and proportions with 95% confidence intervals for pertinent demographic, tobacco use, and reproductive health variables. To test hypotheses for each aim, we conducted multivariable logistic regression models with infant death (delivery through 1 year) as the dependent variable and main effects for tobacco use (ever/never, intensity) of ‘smoked tobacco only during the pregnancy’, ‘smokeless tobacco product use only during pregnancy’, and ‘poly-tobacco use during pregnancy’. Pertinent confounders selected based on the literature included maternal age at birth, ethnicity, marital status, income, education level, region, and exposure to secondhand smoke^1,2^. These confounders were tested individually and in a multivariable model through a typical change of estimate approach (i.e. confounder changes the main effect by 10% or more). We note that these confounders were highly correlated, poverty-related measures, and this poses a challenge for interpreting associations with the main effect variables. To account for this, we also controlled for confounding using well-known propensity score methods described by Austin et al.^23^. Briefly, we created a propensity score variable from the logistic probability function of a logistic model where tobacco use was the outcome and the confounders were included as covariables. The single term for the resulting propensity score was then used to control for multiple confounders in models relating infant death to tobacco.

Statistical analyses were conducted using survey modules of SAS software (version 9.4; SAS Institute Inc. Cary, NC, USA) and SUDAAN software release 9.0 (RTI International, Research Triangle Park, NC). A two-tailed test of statistical significance was used with an alpha of 0.05 for all analyses.

## RESULTS

The NATSC sample we studied consisted of 15998 births (unit of analysis) among 5342 mothers who, at the time of the survey, had a mean age of 44.3 years and had been pregnant at least once. Each mother had birthed a mean of 3 children (range: 1–14) and had an average age of 28.1 years (range: 15–50) at the time of birth. Among these births, the prevalence of tobacco use during pregnancy was 89.8% for never users (smoked or smokeless), 3.3% for exclusive users of smoked tobacco, 6.6% for exclusive users of smokeless tobacco, and 0.2% for poly-tobacco users (smoked + smokeless).

### Birth demographics and tobacco exposure

By demographics, the births were characterized as being to mothers who were of Khmer ethnicity (98.01%), practiced Buddhism (98.01%), currently married (83.23%), had six years or less of education (81.66%), earned less than 1 US$ per day (91.45%), and resided in rural areas (83.36%). The most prevalent occupations for the birth mothers were farming, agriculture, or livestock handling.

Table 1 presents the prevalence (at the level of births) of maternal demographics by patterns of maternal tobacco use. Comparison of non-tobacco users with tobacco users during pregnancy (exclusive smokers, exclusive smokeless users, and poly-tobacco users) indicated important differences across demographic and socioeconomic attributes. Tobacco-using mothers during pregnancy were generally older, had lower income, and less education, particularly the poly-tobacco users, all of whom resided in rural areas. Specifically, exclusive smokers were older (mean age=30.70 years, 95% CI: 29.47–31.94) than non-smoking mothers, and exclusive smokeless tobacco users constituted the oldest subgroup (mean age=33.20 years, 95% CI: 32.55–33.85). Poly-tobacco users presented with the highest proportion of low-income and limited education, with all reporting less than 1 US$ daily income and education of 0–6 years. Indoor smoke exposure was more common in households of exclusive smoking or smokeless mothers (67.63% and 61.21%) and poly-tobacco users (59.31%).

**Table 1:** Selected demographic characteristics by tobacco use patterns among pregnant women enrolled in a national survey of adults in Cambodia (N=5342)

*Characteristics*	*All* *% (95% CI)*	*Non-user* *% (95% CI)*	*Smoke only* *% (95% CI)*	*Chewing only* *% (95% CI)*	*Poly-tobacco user* *% (95% CI)*
**Mean age at birth** (years), mean (95% CI)	28.06 (27.89–28.23)	27.56 (27.39–27.73)	30.70 (29.47–31.94)	33.20 (32.55–33.85)	31.76 (24.56–28.95)
**Ethnicity**					
Khmer	97.41 (95.96–98.35)	97.78 (96.14–98.74)	84.21 (0.33–15.47)	98.70 (96.01–99.58)	96.43 (76.91–99.55)
Cham	1.32 (0.60–2.90)	1.45 (0.65–3.17)	0.33 (0.10–1.02)	0.79 (0.13–4.61)	
Other	1.26 (0.88–1.81)	0.77 (0.46–1.29)	15.47 (10.59–22.03)	0.52 (0.21–1.26)	3.57 (0.45–23.09)
**Religion**					
Buddhist	98.01 (96.57–98.85)	98.31 (96.63–99.16)	87.37 (80.99–91.82)	98.72 (95.94–99.60)	96.43 (76.91–99.55)
Muslim	1.20 (0.52–2.76)	1.30 (0.56–3.01)	0.11 (0.11–0.77)	0.97 (0.22–4.26)	
Christian	0.04 (0.01–0.19)	0.04 (0.01–0.25)			
Other	0.64 (0.43–0.94)	0.33 (0.17–0.62)	9.70 (6.30–14.65)	0.31 (0.10–0.90)	3.57 (0.45–23.09)
None	0.12 (0.06–0.25)	0.03 (0.01–0.10)	2.82 (1.19–6.55)		
**Marital status**					
Currently married	83.23 (81.77–84.60)	85.02 (83.54–86.40)	79.23 (71.45–85.32)	71.35 (64.93–77.01)	65.86 (30.62–89.40)
Separated	2.16 (1.70–2.73)	2.15 (1.69–2.73)	1.86 (0.61–5.51)	2.13 (0.84–5.27)	
Divorced	2.07 (1.70–2.53)	2.18 (1.77–2.69)	1.38 (0.50–3.74)	1.60 (0.84–3.03)	
Widower/widow	11.01 (9.86–12.28)	9.25 (8.14–10.50)	12.24 (7.29–19.84)	24.07 (19.09–29.97)	34.14 (10.60–69.38)
Living together	1.52 (1.11–2.09)	1.39 (0.99–1.95)	5.30 (2.79–9.83)	0.85 (0.25–2.90)	
**Education level** (years)					
0–6	81.66 (79.78–83.40)	80.35 (78.35–82.21)	89.32 (81.90–93.92)	93.37 (88.16–96.39)	100
7–12	17.54 (15.84–19.38)	18.81 (17.01–20.76)	10.45 (5.87–17.91)	5.71 (2.93–10.84)	
>12	0.80 (0.56–1.13)	0.83 (0.60–1.16)	0.23 (0.03–1.64)	0.91 (0.20–4.02)	
**Daily income** (US$)					
<1	91.45 (89.96–92.74)	91.17 (89.59–92.52)	92.49 (84.47–96.54)	93.01 (86.88–96.40)	100
1–2	2.81 (2.22–3.56)	2.80 (2.21–3.54)	1.80 (0.28–10.66)	3.82 (1.65–8.56)	
>2 to 3	2.23 (1.73–2.86)	2.31 (1.77–3.01)	0.43 (0.10–1.78)	2.72 (1.07–6.75)	
>3	3.50 (2.69–4.55)	3.72 (2.82–4.89)	5.28 (2.15–12.42)	0.45 (0.13–1.55)	
**Occupation**					
Does not work	14.83 (13.42–16.37)	14.33 (12.79–16.02)	8.20 (4.11–15.71)	20.26 (15.54–25.96)	32.77 (9.31–69.83)
Professional	0.53 (0.35–0.80)	0.57 (0.37–0.89)	0.34 (0.05–2.41)	0.13 (0.02–0.91)	
Health professional	0.21 (0.12–0.37)	0.23 (0.12–0.41)	0.13 (0.02–0.94)		
Nurse/midwife	0.06 (0.02–0.15)	0.05 (0.02–0.15)		0.19 (0.02–1.34)	
Traditional/faith healer	0.12 (0.03–0.54)	0.10 (0.03–0.42)	0.99 (0.14–6.49)		
Technical	0.61 (0.42–0.88)	0.70 (0.48–1.01)			
Clerical	0.07 (0.03–0.16)	0.08 (0.04–0.18)			
Service	0.36 (0.19–0.66)	0.39 (0.20–0.74)	0.13 (0.03–0.53)		
Fireman/police	0.06 (0.02–0.14)	0.07 (0.03–0.16)			
Sales	11.68 (10.10–13.47)	12.27 (10.53–14.24)	5.27 (2.62–10.35)	6.47 (3.90–10.53)	
Tobacco farming/preparation	0.15 (0.05–0.47)	0.17 (0.06–0.54)			
Farming, agriculture, livestock	65.35 (62.53–68.07)	65.21 (62.19–68.11)	73.82 (63.06–82.33)	69.65 (63.19–75.41)	60.32 (26.18–86.69)
Labor	3.67 (2.77–4.75)	3.44 (2.55–4.63)	9.68 (4.80–18.57)	1.99 (1.06–3.67)	6.92 (0.89–38.00)
Trades and crafts	2.31 (1.74–3.05)	2.34 (2.55–4.63)	1.43 (0.29–6.69)	1.32 (0.47–3.67)	
Armed forces	0.04 (0.01–0.31)	0.05 (0.01–0.36)			
**Residence**					
Urban	16.64 (13.56–20.25)	17.69 (14.47–21.44)	12.61 (5.64–25.84)	6.68 (3.99–10.99)	
Rural	83.36 (79.75–86.44)	82.31 (78.56–85.53)	87.39 (74.16–94.36)	93.32 (89.01–96.01)	100
**Indoor smoke exposure**					
Allowed	45.31 (42.51–48.14)	42.25 (39.37–45.20)	67.63 (57.63–75.98)	61.21 (53.97–67.99)	59.31 (27.24–85.02)
Sometimes allowed	17.81 (15.88–19.91)	17.66 (15.64–19.87)	29.65 (21.13–39.86)	16.34 (11.73–22.29)	31.63 (10.34–64.99)
Never allowed	36.88 (34.06–39.80)	40.09 (37.10–43.15)	2.72 (1.01–7.14)	22.45 (17.01–29.03)	9.06 (1.88–34.15)

### Associations between maternal tobacco exposure and infant mortality

Table 2 presents the adjusted odds ratios for infant mortality across three distinct models of maternal tobacco exposure during pregnancy. After adjusting for maternal age (Model 1), exclusive smokeless and poly-tobacco users exhibited significantly increased odds of infant mortality. Exclusive smokeless tobacco users exhibited approximately two-fold increased odds (adjusted odds ratio, AOR=2.08; 95% CI: 1.15–3.76, p=0.015) compared to non-users. The odds increased among poly-tobacco users to approximately 5.68-fold (AOR=5.68; 95% CI: 1.03–31.46, p=0.047). Meanwhile, exclusive smokers had a higher odds ratio than non-users, but it did not reach statistical significance (AOR=1.72; 95% CI: 0.51–5.79, p=0.38).

**Table 2:** Multivariable models examining associations between tobacco use during pregnancy and infant mortality among pregnant women enrolled in a national survey of adults in Cambodia (5342)

*Variables*	*Model 1*		*Model 2*		*Model 3*	
	*AOR (95% CI)*	*p*	*AOR (95% CI)*	*p*	*AOR (95% CI)*	*p*
**Tobacco exposure patterns during pregnancy**						
Never user (reference)	1		1		1	
Exclusive smoker of tobacco	1.72 (0.51–5.79)	0.377	1.73 (0.50–6.07)	0.383	1.70 (0.49–5.92)	0.403
Exclusive smokeless user of tobacco	2.08 (1.15–3.76)	0.015	2.04 (1.11–3.74)	0.022	2.00 (1.09–3.69)	0.026
Poly-tobacco user (smoked and smokeless)	5.68 (1.03–31.46)	0.047	5.54 (1.02–30.06)	0.047	5.39 (1.00–29.17)	0.050

AOR: adjusted odds ratio. Model 1: adjusted for age at pregnancy only. Model 2: adjusted for age at pregnancy, ethnicity, marital status, income, education level, and region (urban/rural). Model 3: adjusted as in Model 2 but including secondhand smoke exposure.

We note that the odds ratios for poly-tobacco use and exclusive smokers were based on less than ten events. These trends remained even after adjusting for additional demographic covariates (Models 2 and 3), such as ethnicity, marital status, income, education level, region, and secondhand smoke exposure. Exclusive smokeless tobacco users maintained a similar adjusted odds ratio of 2.04 (95% CI: 1.11–3.74, p=0.022), and poly-tobacco users continued to display a markedly elevated risk, with an adjusted odds ratio of 5.54 (95% CI: 1.02–30.06, p=0.047). Further adjustment for secondhand smoke did not alter these trends (Model 3).

### Influence of smokeless tobacco ingredients on infant mortality

**Figure 1:**
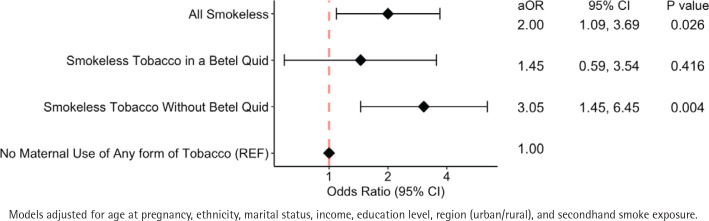
Multivariable models examining the association between smokeless tobacco use and infant mortality among pregnant women enrolled in a national survey of adults in Cambodia (N=5342)

**Figure 2:**
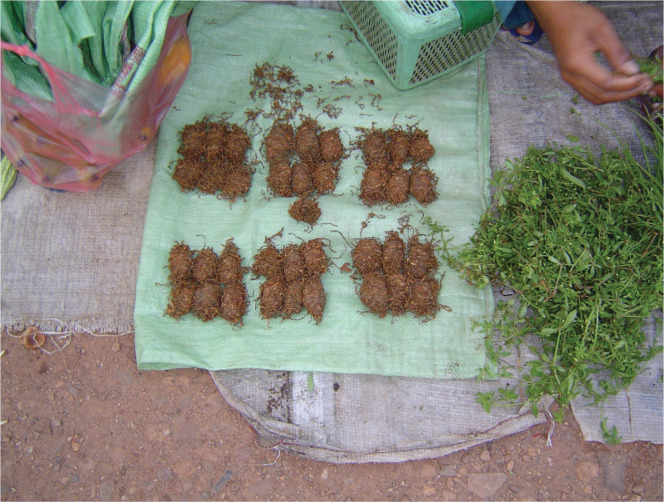
Smokeless tobacco for chewing in rural Asia (the picture was collected during qualitative research on smokers in Cambodia and used in picture cards that accompany survey items)

We conducted an analysis to investigate further the impact of adding other ingredients (areca nut, areca leaf, slaked lime) to the smokeless tobacco that is chewed as a quid during pregnancy (Figure 1). We found that even those pregnant women who chewed a quid with only tobacco leaves (i.e. no addition of areca nut, areca leaf, or slaked lime) experienced a three-fold increase in the odds of infant mortality (AOR=3.05, 95% CI: 1.45–6.45, p=0.004) after adjusting for age at pregnancy, ethnicity, marital status, income, education level, region, and secondhand smoke exposure (Figure 1). This type of quid of unprocessed tobacco is depicted in Figure 2. For pregnant women who chewed quid that included the betel quid ingredients (tobacco, areca nut, areca leaf, and slaked), a 45% increase in odds was observed (AOR=1.45; 95% CI: 0.59–3.54), that did not reach statistical significance (p=0.42) (Figure 1).

## DISCUSSION

There is very convincing evidence that the smoking of combustible cigarettes by pregnant women is a risk factor for fetal and neonatal death^2,24,25^. This evidence has led to clinical, public health, or cultural measures to help women quit or avoid smoking tobacco during pregnancy, through either total abstinence from all tobacco products or by only using a non-combustible tobacco product as a cessation aid (i.e. e-cigarette, nicotine replacement therapy) or cultural practice (i.e. snus, iqmik, betel quid). In some traditional medicine and midwife practices in rural Cambodia, our group has shown that smokeless tobacco is used by pregnant women for relief of morning sickness^11-14^. Our study of a national sample of Cambodian women has the strength that the only smokeless tobacco product available was chewing tobacco in the form of a quid that often included other traditional betel quid components used throughout Asia, such as areca nut, areca leaf, and slaked lime.

Our major findings from Cambodia indicate that a significant, more than two-fold increase in odds of infant mortality was associated with the following habits in pregnant women: 1) exclusive smokeless tobacco use in the form of a tobacco quid that was chewed, and 2) poly-tobacco (smoked + smokeless) use. A further look at the betel quid survey items indicated that even when pregnant women chewed quid with only tobacco leaves (Figure 2), there remained a significant three-fold increase in odds of infant death. For smoked tobacco, we found a 70% increase in odds of infant death for prenatal use that did not attain significance. This may be due to the very low prevalence (<3%) of women smoking cigarettes in Cambodia and that traditional forms of smoking tobacco among women in rural areas (pipes, waterpipes) have declined during the past two decades due to urbanization. Our 2013 report^12^ did show that pregnant women in Cambodia smoking cigarettes and traditional pipes experience significantly higher odds of infant death.

### Does smokeless tobacco use by pregnant women increase their risk of adverse pregnancy outcomes?

Our findings raise the possibility that when a pregnant person chews tobacco leaves with no processing other than washing in water and drying (Figure 2), there is a significant increase in the odds of infant death. These findings need further investigation in prospective designs.

We note that our findings are concordant with data on maternal smokeless tobacco use from several nations. In India, an early cohort study of 1217 women linked maternal use of smokeless tobacco products during pregnancy to a significant 2.6-fold increase in stillbirth risk^26^. Specific smokeless tobacco products, such as mishri (powdered tobacco used orally, sometimes as a dentifrice) and gutka (commercially produced betel quid) were associated with significant 2.5-fold and 5.5-fold increases in risk, respectively^26^. In the US, a 2021 report^15^ from 72056 pregnant women in the PRAMS study reveals that for exclusive users of e-cigarettes, there was a significant increase in the prevalence of low birth weight (adjusted prevalence ratio=1.88; 95% CI: 1.38–2.57) and preterm birth (adjusted prevalence ratio=1.69; 95% CI 1.20–2.39). In a study of 2061514 births from the Swedish National Birth Registry, pregnant women using snuff during pregnancy experienced a significant 71% increase in odds of neonatal mortality and a three-fold increase in odds of Sudden Infant Death Syndrome^27^. Our work adds a new insight that, for pregnant women, chewing of smokeless tobacco with no processing or additive is associated with higher rates of infant death.

### Mechanisms of smokeless tobacco use by pregnant women producing adverse birth outcomes

There is emerging evidence that maternal/fetal exposure to nicotine without combustion or additives can adversely affect the development and survival of the fetus. Nicotine exposure during pregnancy exerts its detrimental effects primarily through the induction of vasoconstriction in uteroplacental blood vessels, thereby reducing placental blood flow, oxygen, and nutrient delivery, leading to potential hypoxic conditions and impaired fetal development^28,29^. Additionally, nicotine exposure during critical periods of fetal development has been linked to epigenetic modifications that alter gene expression in vital organs, including the heart, lungs, and brain, with lasting effects on health and disease susceptibility^30,31^. Our recent studies using pregnant rat models have revealed that both e-cigarette aerosol vaping and nicotine exposure during pregnancy lead to fetal programming of neurovascular and cardiovascular dysfunction in postnatal life. This programming occurs through epigenetic regulatory mechanisms, which involve alterations in DNA methylation, RNA methylation, and miRNA expression^32-34^.

Our work showed a non-significant 45% increase in odds of infant death was associated with a pregnant woman in Cambodia chewing a betel quid with tobacco, areca nut, areca leaf, and slaked lime. The effect of areca nut, areca leaf, and slaked lime in this type of betel quid is unknown. It is noteworthy that the areca nut and leaf do contain plant-based alkaloids (arecaidine, arecoline, guvacine, and guvacoline) that are cholinergic agonists like nicotine. Mutagenic effects of these alkaloids have been shown in animal models. One potential explanation for the higher rate of infant death for tobacco-only quid products is that they have more tobacco than betel quid products with the areca nut/leaf. In previous studies of Cambodian women, our group has reported that those chewing smokeless tobacco exhibited cotinine levels that were comparable to male smokers^20^.

### Implications for pregnant women who are poly-tobacco users during pregnancy

Our data indicated that a significant five-fold increase in the odds of infant death was associated with poly-tobacco use among pregnant women in Cambodia. We note from our data from several surveys that the rate of smoking cessation in Cambodia is <1%. Thus, we do not anticipate that any of the poly-tobacco users in the sample we studied were attempting smoking cessation by transitioning to some use of poly-tobacco use. We note that nicotine metabolism increases during pregnancy and it may be that pregnant women in Cambodia have increased tobacco use to the point of using all forms of available household tobacco (smokeless and smoked) to avoid nicotine withdrawal symptoms.

Our finding of particularly high risk of adverse pregnancy outcomes due to poly-tobacco (smokeless and smoked) use is concordant with data from several nations. In the US, nationwide PRAMS data indicated that women who used both cigarettes and e-cigarettes during late pregnancy had increased odds of small for gestational age (SGA) infants (OR=2.3; 95% CI: 1.3–4.1)^35^. It is noteworthy that the poly-tobacco use harm in the PRAMS study was not lower than the exclusive use of combustible cigarettes (OR=1.7; 95% CI: 1.1–2.7)^36^. Thus, from the PRAMS study, there is no evidence that transitioning to e-cigarettes necessarily reduces the harm of adverse birth outcomes.

### Limitations

There are several limitations of our study that need discussion. First, similar to most national health surveys in the Western Pacific Region, we are using interviewer-reported data for tobacco use and birthing history. In Cambodian adults, we have validated these smoked and smokeless tobacco items against salivary cotinine^20^, and found correlation coefficients (questionnaire measures correlated with salivary cotinine) of >0.40. For the birthing history items, we used a birthing history survey that was developed for the Cambodian Demographic and Health Survey and has been used for national surveys for the past 20 years. Secondly, the measurement units used for age at birth and age at tobacco cessation/initiation were years, introducing some measurement error in the maternal tobacco use during pregnancy measures. Furthermore, the NATSC 2012 data do not account for e-cigarette use, though its prevalence in Cambodia remains relatively low, accounting for <0.1% of the population in 2021, and it is also not a legal product. Finally, while we were able to assess less-than-daily usage for current users, these data were unavailable for former users, thereby limiting our ability to examine the effect of less-than-daily usage across birthing history.

When considering causality, we note the limitation that these findings are from a cross-sectional design study and need further investigation using a prospective design to test whether prior exposure is associated with infant death. It is noteworthy that we are relating prior recalled exposure (i.e. based on self-reported age at initiation of tobacco) and thus have some temporal separation of exposure and disease. Also, we have not controlled for all possible confounders and thus some residual confounding remains. Generalizing these findings outside of rural Asia is also limited and more research is needed on the effect of pregnant women using smokeless products in the context of high-income countries. Despite these limitations, our study provides significant evidence to support further prospective investigation into the relationship between poly-tobacco use and adverse birth outcomes.

## CONCLUSIONS

Among pregnant women in Cambodia, we found that several patterns of smokeless tobacco use were associated with an increase in the odds of infant death. We found that even when pregnant women limited their habit of chewing tobacco leaves with no processing or additives, there remained higher odds of fetal or infant death from that pregnancy. Our findings raise the possibility that use of smokeless tobacco products during pregnancy to reduce harm to the fetus may not protect against the risk of infant death. These trends need further investigation in prospective studies.

## Data Availability

The data supporting this research are available from the authors on reasonable request.

## References

[1] Salihu HM, Wilson RE. Epidemiology of prenatal smoking and perinatal outcomes. Early Hum Dev. 2007;83(11):713-720. doi:10.1016/j.earlhumdev.2007.08.002 17884310

[2] Pineles BL, Hsu S, Park E, Samet JM. Systematic review and meta-analyses of perinatal death and maternal exposure to tobacco smoke during pregnancy. Am J Epidemiol. 2016;184(2):87-97. doi:10.1093/aje/kwv301 27370789 PMC4945701

[3] Knopik VS, Maccani MA, Francazio S, McGeary JE. The epigenetics of maternal cigarette smoking during pregnancy and effects on child development. Dev Psychopathol. 2012;24(4):1377-1390. doi:10.1017/S0954579412000776 23062304 PMC3581096

[4] Bloch M, Althabe F, Onyamboko M, et al. Tobacco use and secondhand smoke exposure during pregnancy: an investigative survey of women in 9 developing nations. Am J Public Health. 2008;98(10):1833-1840. doi:10.2105/AJPH.2007.117887 18309125 PMC2636473

[5] Lange S, Probst C, Rehm J, Popova S. National, regional, and global prevalence of smoking during pregnancy in the general population: a systematic review and meta-analysis. Lancet Glob Health. 2018;6(7):e769-e776. doi:10.1016/S2214-109X(18)30223-7 29859815

[6] Glover M, Phillips CV. Potential effects of using noncombustible tobacco and nicotine products during pregnancy: a systematic review. Harm Reduct J. 2020;17(1):16. doi:10.1186/s12954-020-00359-2 32122384 PMC7053110

[7] Hajek P, Przulj D, Pesola F, et al. Electronic cigarettes versus nicotine patches for smoking cessation in pregnancy: a randomized controlled trial. Nat Med. 2022;28(5):958-964. doi:10.1038/s41591-022-01808-0 35577966 PMC9117131

[8] Rygh E, Gallefoss F, Grøtvedt L. Trends in maternal use of snus and smoking tobacco in pregnancy. A register study in southern Norway. BMC Pregnancy Childbirth. 2019;19(1):500. doi:10.1186/s12884-019-2624-9 31842873 PMC6915947

[9] Dahlin S, Gunnerbeck A, Wikström AK, Cnattingius S, Edstedt Bonamy AK. Maternal tobacco use and extremely premature birth - a population-based cohort study. BJOG. 2016;123(12):1938-1946. doi:10.1111/1471-0528.14213 27411948

[10] Patten CA, Windsor RA, Renner CC, et al. Feasibility of a tobacco cessation intervention for pregnant Alaska Native women. Nicotine Tob Res. 2010;12(2):79-87. doi:10.1093/ntr/ntp180 20018946 PMC2816194

[11] Singh PN, Yel D, Sin S, et al. Tobacco use among adults in Cambodia: evidence for a tobacco epidemic among women. Bull World Health Organ. 2009;87(12):905-912. doi:10.2471/BLT.08.058917 20454481 PMC2789360

[12] Singh PN, Eng C, Yel D, Kheam T, Job JS, Kanal K. Maternal use of cigarettes, pipes, and smokeless tobacco associated with higher infant mortality rates in Cambodia. Asia Pac J Public Health. 2013;25(5 Suppl):64S-74S. doi:10.1177/1010539513493458 PMC5043076 24092813

[13] Singh PN, Kheam T, Lopez J, Job JS, Yel D. Patterns of maternal tobacco use among Cambodian women: findings from a nationwide sample. Asia Pac J Public Health. 2013;25(5 Suppl):54S-63S. doi:10.1177/1010539513487014 23666842

[14] Singh PN, Natto Z, Yel D, Job J, Knutsen S. Betel quid use in relation to infectious disease outcomes in Cambodia. Int J Infect Dis. 2012;16(4):e262-e267. doi:10.1016/j.ijid.2011.12.006 22296863 PMC3307941

[15] Regan AK, Bombard JM, O’Hegarty MM, Smith RA, Tong VT. Adverse birth outcomes associated with prepregnancy and prenatal electronic cigarette use. Obstet Gynecol. 2021;138(1):85-94. doi:10.1097/AOG.0000000000004432 34259468 PMC10896115

[16] Brinchmann BC, Vist GE, Becher R, et al. Use of Swedish smokeless tobacco during pregnancy: a systematic review of pregnancy and early life health risk. Addiction. 2023;118(5):789-803. doi:10.1111/add.16114 36524899

[17] De Silva M, Panisi L, Brownfoot FC, et al. Systematic review of areca (betel nut) use and adverse pregnancy outcomes. Int J Gynaecol Obstet. 2019;147(3):292-300. doi:10.1002/ijgo.12971 31520411

[18] Ferry LH, Job J, Knutsen S, et al. Mentoring Cambodian and Lao health professionals in tobacco control leadership and research skills. Tob Control. 2006;15 Suppl 1(Suppl 1):i42-i47. doi:10.1136/tc.2005.015008 16723675 PMC2563552

[19] Banta JE, Addison A, Job JS, Yel D, Kheam T, Singh PN. Patterns of alcohol and tobacco use in Cambodia. Asia Pac J Public Health. 2013;25(5 Suppl):33S-44S. doi:10.1177/1010539512464649 23165486 PMC5043075

[20] Singh PN, Khieng S, Yel D, Nguyen D, Job JS. Validity and reliability of survey items and pictograms for use in a national household survey of tobacco use in Cambodia. Asia Pac J Public Health. 2013;25(5 Suppl):45S-53S. doi:10.1177/1010539513486920 23695538

[21] Um S, Sopheab H. The factors associated with under-five mortality in Cambodia: data analysis of the Cambodia demographic and health survey. Cambodia Journal of Public Health. 2021;2:12. Accessed July 27, 2024. https://cjph.niph.org.kh/index.php/cjph/article/view/155/31

[22] Lohr SL. Sampling: Design and Analysis. 2nd ed. Chapman and Hall/CRC; 2019. doi:10.1201/9780429296284

[23] Austin PC. An introduction to propensity score methods for reducing the effects of confounding in observational studies. Multivariate Behav Res. 2011;46(3):399-424. doi:10.1080/00273171.2011.568786 21818162 PMC3144483

[24] U.S. Office on Smoking and Health. Women and Smoking: A Report of the Surgeon General. U.S. Centers for Disease Control and Prevention; 2001. Accessed July 27, 2024. https://www.ncbi.nlm.nih.gov/books/NBK44303/

[25] Cnattingius S, Haglund B, Meirik O. Cigarette smoking as risk factor for late fetal and early neonatal death. BMJ. 1988;297(6643):258-261. doi:10.1136/bmj.297.6643.258 3416144 PMC1833960

[26] Gupta PC, Subramoney S. Smokeless tobacco use and risk of stillbirth: a cohort study in Mumbai, India. Epidemiology. 2006;17(1):47-51. doi:10.1097/01.ede.0000190545.19168.c4 16357594

[27] Gunnerbeck A, Lundholm C, Rhedin S, et al. Association of maternal snuff use and smoking with Sudden Infant Death Syndrome: a national register study. Pediatr Res. 2023;94(2):811-819. doi:10.1038/s41390-022-02463-4 36755185 PMC10382311

[28] Bruin JE, Gerstein HC, Holloway AC. Long-term consequences of fetal and neonatal nicotine exposure: a critical review. Toxicol Sci. 2010;116(2):364-374. doi:10.1093/toxsci/kfq103 20363831 PMC2905398

[29] Wickström R. Effects of nicotine during pregnancy: human and experimental evidence. Curr Neuropharmacol. 2007;5(3):213-222. doi:10.2174/157015907781695955 19305804 PMC2656811

[30] Chhabra D, Sharma S, Kho AT, et al. Fetal lung and placental methylation is associated with in utero nicotine exposure. Epigenetics. 2014;9(11):1473-1484. doi:10.4161/15592294.2014.971593 25482056 PMC4623268

[31] Gould TJ. Epigenetic and long-term effects of nicotine on biology, behavior, and health. Pharmacol Res. 2023;192:106741. doi:10.1016/j.phrs.2023.106741 37149116

[32] Jian J, Zhang P, Li Y, et al. Reprogramming of miR-181a/DNA methylation patterns contribute to the maternal nicotine exposure-induced fetal programming of cardiac ischemia-sensitive phenotype in postnatal life. Theranostics. 2020;10(25):11820-11836. doi:10.7150/thno.48297 33052248 PMC7546014

[33] Walayat A, Li Y, Zhang Y, et al. Fetal e-cigarette exposure programs a neonatal brain hypoxic-ischemic sensitive phenotype via altering DNA methylation patterns and autophagy signaling pathway. Am J Physiol Regul Integr Comp Physiol. 2021;321(5):R791-R801. doi:10.1152/ajpregu.00207.2021 34524928 PMC8616627

[34] Liu B, Xia L, Li Y, et al. Prenatal nicotine exposure raises male blood pressure via FTO-mediated NOX2/ROS signaling. Hypertension. 2024;81(2):240-251. doi:10.1161/HYPERTENSIONAHA.123.21766 37795601 PMC10873091

[35] Wang X, Lee NL, Burstyn I. Smoking and use of electronic cigarettes (vaping) in relation to preterm birth and small-for-gestational-age in a 2016 U.S. national sample. Prev Med. 2020;134:106041. doi:10.1016/j.ypmed.2020.106041 32105682

[36] Cardenas VM, Ali MM, Fischbach LA, Nembhard WN. Dual use of cigarettes and electronic nicotine delivery systems during pregnancy and the risk of small for gestational age neonates. Ann Epidemiol. 2020;52:86-92.e2. doi:10.1016/j.annepidem.2020.08.002 32805398

